# The hidden toll of community outreach

**DOI:** 10.7554/eLife.85166

**Published:** 2022-12-01

**Authors:** Raul A Ramos

**Affiliations:** 1 Miller Institute for Basic Research in Science, University of California, Berkeley Berkeley United States

**Keywords:** research culture, sparks of change, equity, diversity, inclusion, outreach

## Abstract

Caught in a system eager for success stories, a PhD student from an underrepresented background learns how to balance his challenges in the lab with his desire to serve his community.

My first panic attack occurred in 2020, two weeks before the initial COVID-19 lockdown. I was at a seminar when, without warning, I felt like I couldn't breathe. Worried that I would lose my lunch then and there, I exited the room promptly. Many more episodes would follow over the next few months, including some in my sleep. Every time they happened, the triggers were the same: lecture halls, attending a presentation, or the idea of giving one. The irony of these triggers was not lost on me; I had spent the past few years heavily involved in outreach, delivering talk after talk in schools across the country. I realize now that I'd fallen into the pitfalls of a system which capitalized on this work without considering the pressure it would create for me. To move forward, I knew I had to find a way to stay true to myself and reconcile helping my community with trying to become a research scientist.

**Figure 1:**
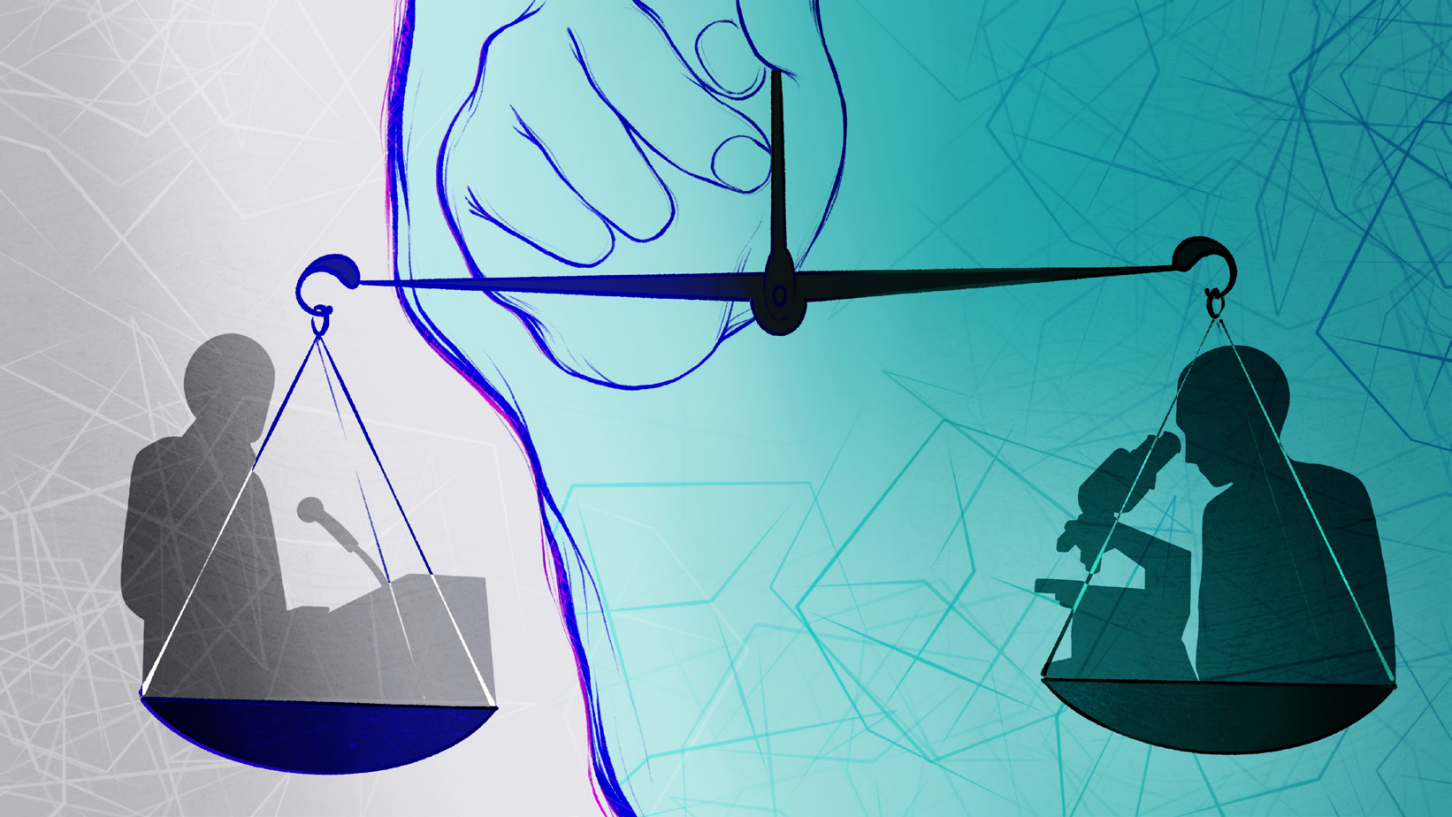
Devoted to giving back to his community, a PhD student from an underrepresented background faces difficulties in the lab while being heralded as a success story.

Image credit: Marzia Munafò; @munafomarzia (CC BY 4.0)

Three years earlier and barely two years into my PhD, I received an email with the subject line: ’Want to inspire students to study science?‘. The Association for the Advancement of Science (AAAS) was looking for academics ’interested in sharing with students how they fell in love with science, obstacles they've overcome, and why they aren't the stereotypical image of a scientist‘. As a teenager, role models in my community had saved me from a more dangerous path; I felt I was being offered a chance to perhaps be *that* person for someone else. I replied enthusiastically to the email, and my outreach efforts began there.

Call me naïve, but I was unprepared for how much academic institutions love a good story – and mine was definitely a hit. As a teenager, I was expelled from middle school and then incarcerated as a juvenile offender. Fast-forward a few years, I was pursuing a PhD in neuroscience at a top research university in the United States. My outreach work gathered momentum more quickly than I had expected. Just one year after my first event, I travelled across the country on behalf of AAAS to engage in a marathon of over 15 presentations in three days. I spoke to about 200 kids, mostly incarcerated students who shuffled to my presentations in handcuffs. Having shuffled in their shoes, I felt a heavy responsibility to continue my outreach efforts. I genuinely believed I could positively impact people’s lives if I continued to invest my energy in these extracurricular activities. And so, I did.

This work brought a level of publicity I had not anticipated. I was the subject of several online articles, and my university contacted me for a magazine feature. I even appeared on a postcard for donors, being quite literally milled into donor fodder. There was an eager attitude towards showcasing someone with such a clear redemption arc in their personal story. Regrettably, I was not focused on the consequences this tokenization could have on me or others looking to follow in my footsteps. Instead, I concentrated on leveraging the attention I received to create more opportunities for outreach work.

The success of my outreach events may have suggested that my PhD was going well. But, beneath the surface, I was fighting all the battles of a junior graduate student. I struggled to learn the highly specialized techniques I needed for my work while trying to fit in with my new lab and dealing with a cross-country move which had turned my world upside down. It took some time before I started to recognize the toll that juggling my scientific training and outreach work took on me. It is not until now, a year after defending my PhD, that I can more clearly articulate how overwhelmed I was by the pressure.

I had always wanted to authentically convey my life experiences, but a dissonance emerged. At outreach events, I was taking the stage as someone who had "made it"; back in the lab, I struggled to get my experiments to work. As my confidence began to crack, feelings of imposterhood oozed out. I started to feel like a phony. I never considered halting my outreach activities, believing they were intrinsically more valuable than my work as a scientist. Instead, like many graduate students, I thought about dropping out and I looked into careers that would take me away from the bench and into public service. Ultimately, my mentors convinced me that there was still something to be gained from overcoming my scientific challenges. So, I pushed forward; I muscled on until the weight of these unresolved feelings became too much and the panic attacks started.

I kept my anxieties a secret from all but those closest to me. When the lockdowns brought in-person presentations to a halt, I used the pandemic as a convenient excuse to not engage in any outreach. The truth is, I was learning about my symptoms and how to manage them, attempting to cope with what was essentially an extreme case of late-onset stage fright. For many years I had been caught in a balancing act, trying to juggle two parts of my life that were no longer in phase: I had to balance the scales again. I decided to recommit myself to my scientific training. I used this time to grow as a scientist and I finished my PhD, reaching a stage where I could finally transcend the basics to enter more creative territories. This was when I received an invitation to give an outreach talk at my undergraduate alma mater.

Suddenly I was in a familiar place, standing at the front of the classroom where I had taken general chemistry ten years ago. I had given this talk a dozen times before but never as Dr. Ramos and never at the institution where my journey into science began. This was a homecoming, an inauguration, and a victory lap, all woven together by threads of nostalgia and warm memories. I had sat in these students' chairs, and now I was here to talk about how I had made it to the other side of my PhD. I forced myself to focus by making eye contact with my audience, and soon the students picked up on the fact that this was a unique full-circle moment. My presentation transitioned into a lively back-and-forth about my experiences and careers in science. I felt so much pride for these students and for my community. A realization finally dawned on me: I was there to inspire them, but they were, in fact, inspiring me. They had been doing so all along. When growing pains and imposter syndrome had made me look for the door, I doubled down on becoming the scientist and role model I wanted to be for *them*.

Now that the dust has settled, I've begun to unpack my grad school experience. Community outreach is often seen as performing a service, a one-directional act of giving back to inspire and uplift others. I understand now that giving back motivates me to keep growing as a scientist, but that I took on too much too quickly. I've learned that to be a better role model for younger students, I also need to take care of myself professionally and personally, and I am now much stricter when choosing which opportunities I allow myself to get involved with. However, I have also been reflecting on how the system was all too eager to facilitate my outreach efforts without considering how they might impact me.

To be clear, the people I have collaborated with along the way were all well-intentioned, and they provided me with a platform to do meaningful work. Once I found myself working within the system, I leaned into it. I assumed that more publicity would give me more chances to do the work I believed in — and it did. I recognize that I actively participated in my own tokenization, but I am also aware that there was an inherent imbalance of power. As a graduate student trying to build a career, I suspect that institutions knew I would not say no to an opportunity.

This sort of institutional 'propaganda' is not inconsequential. Equity, diversity, and inclusion initiatives are often a labor of love performed mainly by those with marginalized identities. We usually do so without compensation for our time and expertise or even the recognition that this effort can cut into time and energy vital for our scientific development. By becoming our university’s next 'poster person' and participating in our own tokenization, we allow institutions to paint a rosy picture and appear more inclusive and supportive than they actually are.

I firmly believe that there is a continued need for scientists to engage in community outreach, and I hope that others can explore this work while being wary of the system in which they are working. Our younger generations do need role models, but we should never sacrifice who we are along the way.

## Share your experiences

This article is a Sparks of Change column, where people around the world share moments that illustrate how research culture is or should be changing. Have an interesting story to tell? See what we’re looking for and the best ways to get in touch here.

